# The Seroprevalence of Pandemic Influenza H1N1 (2009) Virus in China

**DOI:** 10.1371/journal.pone.0017919

**Published:** 2011-04-21

**Authors:** Cuiling Xu, Tian Bai, A. Danielle Iuliano, Min Wang, Lei Yang, Leying Wen, Yuhong Zeng, Xiaodan Li, Tao Chen, Wei Wang, Ying Hu, Limei Yang, Zi Li, Shumei Zou, Dexin Li, Shiwen Wang, Zijian Feng, Yanping Zhang, Hongjie Yu, Weizhong Yang, Yu Wang, Marc-Alain Widdowson, Yuelong Shu

**Affiliations:** 1 State Key Laboratory for Molecular Virology and Genetic Engineering, National Institute for Viral Infectious Disease Control and Prevention, Chinese Center for Disease Control and Prevention, Beijing, China; 2 Chinese Center for Disease Control and Prevention, Beijing, China; 3 Influenza Division, National Center for Immunization and Respiratory Diseases, Centers for Disease Control and Prevention, Atlanta, Georgia, United States of America; Duke-National University of Singapore Graduate Medical School, Singapore

## Abstract

**Background:**

Mainland China experienced pandemic influenza H1N1 (2009) virus (pH1N1) with peak activity during November-December 2009. To understand the geographic extent, risk factors, and attack rate of pH1N1 infection in China we conducted a nationwide serological survey to determine the prevalence of antibodies to pH1N1.

**Methodology/Principal Findings:**

Stored serum samples (n = 2,379) collected during 2006-2008 were used to estimate baseline serum reactogenicity to pH1N1. In January 2010, we used a multistage-stratified random sampling method to select 50,111 subjects who met eligibility criteria and collected serum samples and administered a standardized questionnaire. Antibody response to pH1N1 was measured using haemagglutination inhibition (HI) assay and the weighted seroprevalence was calculated using the Taylor series linearization method. Multivariable logistic regression analyses were used to examine risk factors for pH1N1 seropositivity. Baseline seroprevalence of pH1N1 antibody (HI titer ≥40) was 1.2%. The weighted seroprevalence of pH1N1 among the Chinese population was 21.5%(vaccinated: 62.0%; unvaccinated: 17.1%). Among unvaccinated participants, those aged 6-15 years (32.9%) and 16-24 years (30.3%) had higher seroprevalence compared with participants aged 25–59 years (10.7%) and ≥60 years (9.9%, P<0.0001). Children in kindergarten and students had higher odds of seropositivity than children in family care (OR: 1.36 and 2.05, respectively). We estimated that 207.7 million individuals (15.9%) experienced pH1N1 infection in China.

**Conclusions/Significance:**

The Chinese population had low pre-existing immunity to pH1N1 and experienced a relatively high attack rate in 2009 of this virus. We recommend routine control measures such as vaccination to reduce transmission and spread of seasonal and pandemic influenza viruses.

## Introduction

On June 11, 2009, the World Health Organization (WHO) declared the first influenza pandemic of the21st century caused by a novel swine-origin influenza A H1N1 virus [1], which contains gene segments derived from classical swine H1N1 virus, human seasonal influenza H3N2 virus, avian influenza H1N1 virus and Eurasian swine H1N1 influenza virus [2].

Studies on the extent of infection with pH1N1 are essential for pandemic severity assessment and for the development of response and vaccination strategies. Modeling methods have been used to estimate the incidence of infection during the pandemic period, using clinical surveillance data in which only patients with influenza-like illness who seek care are captured, while those who do not seek care or have asymptomatic infections are excluded [3]–[5]. These estimates provide useful and timely information, but may lead to an underestimation of the actual number of infections. Therefore, serological studies have been recommended to more accurately estimate the attack rate and the extent of infection of 2009 pandemic influenza A H1N1 (pH1N1) virus infection [6]. Such serological studies have previously been conducted using convenience serum samples [7]–[13]. Miller, et al. estimated that approximately one in every three children in the United Kingdom (UK) had serological evidence of pH1N1 infection which was nearly ten times higher than the estimated incidence of pH1N1 from clinical surveillance [7]. Chen, et al. estimated that 13% of participants from a community cohort of adults in Singapore had serological evidence of pH1N1 infection following the June to September 2009 wave of pH1N1 [8].

On May 11, 2009, the first imported human pH1N1 case was detected in mainland China. Activity for pH1N1 remained low until the end of August, increased sharply in September, and peaked in late November. The purpose of this study was to estimate the baseline cross reactive antibody response to pH1N1 virus prior to introduction of the virus in mainland China using a convenience sample of serum collected during 2006–2008, to estimate the attack rate or seroprevalence of pH1N1 infection after the first wave of pH1N1 infection in January 2010 using a serological study (Figure S1), and to examine factors associated with serological response to pH1N1 infection. We conducted a multi-stage random-sampling serological study to determine the seroprevalence of pH1N1 in mainland China representative of different areas and ages, to understand the geographic extent of infection and to assess risk factors of pH1N1 infection in China. Combining the findings from these two studies, we were able to also estimate the attack rate of pH1N1 infection after the first wave of the pandemic in mainland China.

## Results

### Baseline cross reactive antibody response to pandemic influenza H1N1 (2009) virus

The baseline cross reactive antibody response to pH1N1 infection (HI titer of ≥40) by age group among the convenience sample of 2,379 individuals is shown in Table 1. The overall baseline cross reactive antibody response to pH1N1 infection among the population was 1.2% (95% confidence interval [95% CI]: 0.7–1.6%). Examining the data by age group showed that individuals aged 16–24 had the highest baseline cross reactive antibody response to pH1N1 infection (3.3%) in comparison with individuals in other age categories (0–5 years: 0%, 6–15 years: 1.1%, 25–59 years: 0.6%, ≥60 years: 2.0%).

**Table 1:** Proportion of baseline sera reactive to pandemic influenza H1N1 (2009) virus in each age group, 2006–2008.

Age (years)	No. of Samples tested(n = 2379)	No. of positive samples	Proportion of positive antibody %	95%CI
0–5	436	0	0	0–0.7
6–15	556	6	1.1	0.4–2.3
16–24	360	12	3.3	1.7-5.8
25–59	534	3	0.6	0.1–1.6
60–	493	10	2.0	1.0–3.7

### Characteristics of study population of the cross-sectional seroprevalence study

In January 2010 we enrolled 50,458 subjects in the cross-sectional study. Of those, 50,403 blood samples were collected and 50,350 participants completed both the questionnaire and blood sample collection. Of these, 239 subjects were excluded because of missing demographic data (n = 161) or having insufficient serum sample (n = 78), leaving data from 50,111 (99.5%) subjects for analysis, Demographic characteristics of the entire sample are shown in Table 2. There were no statistically significant differences in the distribution of the data by age group, gender, region, and community setting (capital city, urban area, or rural) between the study subjects and the true Chinese population. There were 7,799 (15.6%) subjects who reported receiving the pH1N1 vaccine compared with 42,300 (84.4%) who reported not receiving the pH1N1 vaccine, and 12 (0.02%) subjects reported unknown vaccination history. Using sampling weight constructed based on the multi-stage random sampling design to adjust for oversampling of certain age groups and community settings, the weighted proportion of the Chinese population estimated to have received pH1N1 vaccine was 9.7%. Among unvaccinated participants, 39.4% were children in kindergarten or students, 7.3% were children in family care, 4.6% were teachers, doctors or nurses while nearly half (48.7%) reported other occupation (Table 2).

**Table 2:** Characteristics of study population in the cross-sectional survey, January 2010.

Demographic Characteristics	Study subjects N(%) (n = 50,111)	Unvaccinated subjectsN(%) (n = 42,300)	True Chinese population per millionN(%) (n = 1,306.3 million)
Age group, years			
0–5	9,914 (19.8%)	9,512 (22.5)	84.9 (6.5)
6–15	10,500 (21.0%)	7,409 (17.5)	197.3 (15.1)
16–24	9,513 (19.0%)	7,485 (17.7)	164.6 (12.6)
25–59	10,684 (21.3%)	8,984 (21.2)	689.7 (52.8)
≥60	9,500 (18.9%)	8,910 (21.1)	169.8 (13.0)
Gender			
Male	24,090 (48.1%)	20,430 (48.3)	659.7 (50.5)
Female	26,021 (51.9%)	21,870 (51.7)	646.6 (49.5)
Occupation^#^			
Children in family care§	3,150 (6.3%)	3088 (7.3)	
Children in kindergarten	7,118 (14.2%)	6763 (16.0)	
Student	14,014 (28.0%)	9871 (23.4)	
Teacher	936 (1.9%)	609 (1.4)	
Doctor or nurse	2,632 (5.3)	1311 (3.2)	
Other	22,174 (44.3%)	20579 (48.7)	
Urban/rural			
Capital city (Municipalities)	16,558 (33.0%)	13321 (31.5)	115 (8.8)
Other urban areas	16,496 (32.9%)	13,791 (32.6)	446.8 (34.2)
Rural areas	17,057 (34.0%)	15,188 (35.9)	744.6 (57.0)
Region			
Eastern	18,314 (36.6%)	15,483 (36.6)	437.6 (33.5)
Central	18,067 (36.0%)	15,276 (36.1)	502.9 (38.5)
Western	13,730 (27.4%)	11,541 (27.3)	365.8 (28.0)
Vaccination of pH1N1*			
Yes	7,799 (15.6%)		
No	42,300 (84.4%)		
Developed a “cold” since May 1,2009			
Yes	23,867 (47.6%)	19,971 (47.2%)	
No	26,244 (52.4%)	22329 (52.8)	

**NOTE.** # 87 participants were missing occupation data, and 79 unvaccinated participants were missing occupation data.

§ Children in family care is defined as the persons aged ≤15 years that are not student or Children in Kindergarten, or did not worked in any organizations or units.

*12 participants reported unknown vaccination history of pH1N1.

### Weighted prevalence of pandemic influenza H1N1 (2009) virus

Among 50,111 study subjects, 14,776 (29.5%) were antibody positive for the pH1N1 virus. Since we employed a multi-stage sampling method we adjusted for age and other factors to calculate a weighted pH1N1 seroprevalence of 21.5% (95% CI: 20.5–22.5) in the whole Chinese population. The weighted prevalence of pH1N1 antibody response for the subjects who reported receiving vaccine was significantly higher 62.0% (95% CI: 58.8–65.3) than subjects who did not report receiving the vaccine 17.1% (95% CI: 16.1–18.0) (p<0.0001).

Among the unvaccinated study population, we found that individuals aged 6–15 years (32.9%) and 16–24 years (30.3%) had the highest weighted prevalence, individuals aged 25–59 years (10.7%) and ≥60 years (9.9%) had the lowest (Table 3). When examining the weighted seroprevalence among the unvaccinated study population, we found that students had the highest weighted seroprevalence (34.9%), followed by children in kindergarten (26.2%), and participants with other occupations (11.1%). Further, among unvaccinated subjects, weighted prevalence of pH1N1 in eastern provinces (15.2%) was statistically significantly lower than the prevalence in both central (18.6%) and western provinces (19.3%). Among the unvaccinated, the weighted prevalence of pH1N1 infection in other urban areas (19.6%) was statistically significantly higher than the weighted prevalence in rural areas (15.8%), and higher than that in capital cities (17.1%) though the difference between capital cities and other urban areas was not.

**Table 3:** Weighted seroprevalence of 2009 pandemic H1N1 virus antibodies by demographic characteristics among subjects who reported not receiving pH1N1 vaccine (N = 42,300).

Demographic Characteristics	Samples tested	Positive samples	pH1N1antibody (%)	Weighted prevalence ofpH1N1 antibody (%)	95% Confidence Interval
Age group, years					
0–5	9512	2550	26.8	24.7	22.4–26.9
6–15	7409	2677	36.1	32.9	30.2–35.6
16–24	7485	2369	31.6	30.3	27.7–32.9
25–59	8984	1255	14.0	10.7	9.5–12.0
≥60	8910	768	8.6	9.9	8.2–11.7
Gender					
Male	20430	4893	24.0	18.2	16.7–19.6
Female	21870	4726	21.6	16.0	14.8–17.2
Occupation^#^					
Children in family care	3088	662	21.4	20.8	17.6–24.1
Children in kindergarten	6763	1967	29.1	26.2	23.0–29.5
Student	9871	3785	38.3	34.9	32.5–37.3
Teacher	609	136	22.3	16.1	7.9–24.4
Doctor or nurse	1311	303	23.1	19.0	13.7–24.4
Other	20579	2751	13.4	11.1	10.1–12.1
Urban/rural					
Capital city (Municipalities)	13321	3052	22.9	17.1	15.7–18.4
Other urban areas	13791	3333	24.2	19.6	18.2–21.1
Rural areas	15188	3234	21.3	15.7	14.4–17.0
Region					
Eastern	15483	3301	21.3	15.2	13.8–16.7
Central	15276	3554	23.3	18.6	17.3–19.8
Western	11541	2764	23.9	19.3	17.7–20.9
Total	42300	9619	22.7	17.1	16.1–18.0

**NOTE.** # 79 unvaccinated participants were missing occupation data.

To control for possible interactions between factors, multivariable logistic regression was used to estimate the odds ratio (OR) and 95% Confidence Interval (95% CI) for factors associated with pH1N1 antibody response among subjects who reported not receiving the pH1N1 vaccine (Table 4). The adjusted odds of seropositivity to pH1N1 infection for the eastern region (OR: 0.80, 95% CI: 0.68–0.93) were statistically significantly lower than the odds of infection in the western region. There was no statistically significant difference in the odds of infection between the central region (OR: 1.02, 95% CI: 0.89–1.18) and the western region. The odds of pH1N1 infection in the rural areas was statistically significantly lower (OR: 0.79, 95% CI: 0.69–0.90) compared with the odds of infection in other urban areas. The odds of pH1N1 infection in capital city areas (OR: 0. 97, 95% CI: 0.83–1.12) were lower than other urban areas, but were not statistically significantly different from those in other urban areas. Children in kindergarten (OR: 1.36, 95% CI: 1.05–1.76) and students (OR: 2. 04, 95% CI: 1.64–2.54) had significantly higher odds of pH1N1 seropositivity than children who were in family care. In contrast, subjects with other occupation (OR: 0.46, 95% CI: 0.37–0.58) had lower odds of pH1N1 antibody response compared with children in family care. The odds of seropositivity were not statistically different by gender (Table 4).

**Table 4:** Adjusted odds ratios and 95% Confidence Intervals of pH1N1 infection among subjects who reported not receiving pH1N1 vaccine (N = 42,300).

Demographic Characteristics	Weighted prevalence ofpH1N1 antibody (%)	Adjusted OR (95% CI)	p-value
Gender			
Male	18.2	1	
Female	16.0	0.91 (0.79–1.04)	0.15
Occupation#			
Children in family care	20.8	1	
Children in kindergarten	26.2	1.36 (1.05–1.76)	0.0003
Student	34.9	2.04 (1.64–2.54)	<0.0001
Teacher	16.1	0.77 (0.41–1.44)	0.37
Doctor or nurse	19.0	0.82 (0.55–1.22)	0.30
Other	11.1	0.46 (0.37–0.58)	<0.0001
Urban/rural			
Capital city (Municipalities)	17.1	0.97 (0.83–1.12)	0.19
Other urban areas	19.6	1	
Rural areas	15.7	0.79 (0.69–0.90)	0.0003
Region			
Eastern	15.2	0.80 (0.68–0.93)	0.0003
Central	18.6	1.02 (0.89–1.18)	0.02
Western	19.3	1	

**NOTE.** # 79 unvaccinated participants were missing occupation data.

### Attack rate of pandemic influenza H1N1 (2009) virus in the Chinese Population

We estimated the attack rate of infection of pH1N1 from May 2009 to January 2010 to be 15.9% (95% CI: 15.3–16.5%) by subtracting the baseline cross reactive antibody response to pH1N1 infection (1.2%) from the estimated seroprevalence of pH1N1 infection from our study (17.1%). The attack rates by age group were 24.7% (0–5 y), 31.8% (6–15 y), 27% (16–24 y), 10.1% (25–59 y) and 8% (60+y). The estimated number of pH1N1 infections was calculated by multiplying the estimated seroincidence of infection (15.9%) by the total population on mainland China (1,306.3 million) to give a total number of pH1N1 cases of 207.7 million.

## Discussion

We aimed to estimate the adjusted seroprevalence of antibodies to pH1N1 among Chinese adults and children, to estimate the total number of persons infected in China, and to understand risk factors for infection among this population. We found that the seropositivity to pH1N1 was 17.1% after excluding individuals who reported receiving the pH1N1 vaccine, but that the baseline pre-pandemic seropositivity percent was 1.2%, giving an attack rate of pH1N1 in the first pandemic wave of 15.9% in the period May 2009 to January 2010. Further, our study showed that the seroprevalence of pH1N1 infection was higher in the central and western regions compared with the eastern region, higher in urban compared to rural areas and higher in school-aged children (6–15 years).

To our knowledge, this is the first time a multi-stage random-sampling serological study to investigate the seroprevalence of pH1N1 in China has been conducted. We used a serological survey to examine the seroprevalence of pH1N1 because of limitations of the clinical surveillance system in capturing true prevalence of pH1N1 infection. The clinical surveillance systematically captures individuals who seek medical care at hospitals that conduct the surveillance. However, it has been reported that many 2009 pandemic influenza H1N1 cases were mild [14]–[16] and many of those with infection may not have sought medical care and would not have been tested for infection. Additionally, studies have suggested that asymptomatic pH1N1 infection may be common [14]. Our serosurvey results suggest that approximately 207.7 million people in mainland China were infected with pH1N1 from May 2009 to January 2010. As of 31 January 2010, 126,449 clinical pH1N1 cases confirmed through respiratory specimens were reported in mainland China [17], implying that each such confirmed case of pH1N1 represented a possible 1,630 infections.

Our study had several limitations. The haemagglutination-inhibition (HI) assay may not be the most sensitive assay to detect low levels of pH1N1 compared to for example microneutralization, and we may have underestimated seropositivity both in the baseline and the serological survey samples. Also though we recorded a high percentage of seropositive persons who did not report symptoms, this may be due to potential recall bias and we were unable to confirm the presence or absence of respiratory symptoms. Lastly, we were not able to confirm receipt or not of pH1N1 vaccine, and it is possible that people reported receipt of different vaccines as pH1N1 vaccine.

Our study showed school-aged population and young adults had the highest attack rates of pH1N1, which is consistent with studies from the UK and Hong Kong [7], [13]. The attack rate of pH1N1(31.8%) among individuals aged 6–15 yrs was lower compared with individuals aged 5–14 yrs (42%) in the UK study and the attack rate among individuals in Hong Kong aged 5–14 years was 43.4%) [7], [13].

Our findings also indicated that 1.2% of the population had baseline cross reactive antibody response to pH1N1 and only 2.0% of adults aged ≥60 years had an antibody response. Unlike other countries such as the United States, the United Kingdom, Germany, and Finland [7], [9], [18], [19], older adults in China had a lower baseline antibody response to the pH1N1 virus. These findings were similar to findings from a serological study conducted in the Guangxi province of China and other studies in Japan and Singapore [8], [20]–[23]. The lower antibody response among older adults in our study could indicate little or no cross reactivity with previous swine-origin influenza A viruses and is consistent with data from Singapore, but not with recent data from Taiwan [8], [11]. The finding that individuals living in Eastern regions had lower seroprevalence of pH1N1 infection compared with individuals living in both Central and Western regions is similar to findings from the Chinese National Surveillance System during peak influenza periods, the hospital based surveillance system that monitors trends of influenza-like illness. One possible reason why the Eastern region experienced lower seroprevalence of infection although this region is more densely populated and includes most of the major cities in the country is that the eastern region had higher economic level, higher education level and more funding for health care than both the central and western regions (expenditure for health care per capita in 2009: eastern, RMB 929.81; central, RMB 753.09; western, RMB 739.64) [25]. Additionally, our results showed that the seroprevalence of pH1N1 infection was higher in urban areas compared to rural areas. We speculate that the higher seroprevalence of pH1N1 infection in urban areas may be related to more frequent social contacts and greater density of population. Our finding of increased pH1N1 seroprevalence in school-aged children is consistent with recent serological studies in other countries, as well as a study conducted in Beijing, China [7], [9], [10], [12], [13]. In our study, school-aged children had higher odds of antibody response to pH1N1 infection compared with children in family care. The observed higher odds of seropositivity may be the result of intense social mixing patterns in schools and kindergartens possibly contributing to transmission.

As of January 20, 2010, approximately 65.6 million people had received pH1N1 vaccine in mainland China [26], which accounted for 5% of the population. In contrast, our study found that 9.7% of the population received the pH1N1 vaccine. One possible explanation for the difference may be that individuals who reported receiving the vaccine may have been more willing to participate in the study or that participants misreported receiving any vaccine. The observed antibody response among study subjects who reported receiving the vaccine was lower (62%) than the antibody positive rate from a clinical study in China. This study reported that 74.5–97.3% of the subjects receiving 15 μg of nonadjuvanted vaccine achieved a HI titer ≥40 by day 21[23], [24]. The percentage of individuals reaching seropositivity in this study was also higher than another study conducted in Beijing between late-November and early-December, 2009, where 14.0% of participants reached seropositivity [12]. Our findings showed the subjects reported receiving vaccine still obtained higher seroprevalence than the general population, who presumably experienced natural infection. One possible reason for the lower seroprevalence among the vaccinated population in our study population was that the interval of time between vaccination date and sample collection which was less than 2 weeks and may not have been enough time to develop antibody response. Another possible reason could be recall bias with self-reported vaccination history or misclassification of vaccine in some individuals may report receiving pH1N1 vaccine, but may have received a different vaccine.

The Chinese population had low pre-existing immunity to pH1N1, but experienced a relatively high attack rate in 2009 of this virus. Our finding of high seroprevalence of pandemic influenza H1N1 (2009) (21.5%) after the first peak in autumn-winter season of 2009–2010 in mainland China may explained further by the theory that sustainable transmission is not likely when a significant change in viral antigens is not acquired. Our study findings help to enhance the understanding of the 2009 pH1N1 virus and provide valuable information for the Chinese authorities to develop a vaccination strategy for the coming influenza season.

## Materials and Methods

### Baseline serological survey

To assess the baseline prevalence of cross-reactive antibody response to 2009 pandemic H1N1, we used 2,379 stored serum samples collected between 2006 and 2008. These samples were collected from five provinces from different regions of mainland China (Guangdong, Hubei, Shandong, Xinjiang, Yunnan, Figure S2) and were divided into 5 age groups (0–5 years, 6–15 years, 16–24 years, 25–59 years, ≥60 years). Sample sizes for the five age groups ranged from 360 to 556 and more than half (51.1%) were male.

### Random-sampling cross-sectional seroprevalence study

#### Study design

In January 2010, a cross-sectional seroprevelance study to estimate the seroprevalence of 2009 pH1N1 virus infection was approved by the Ministry of Health (MoH) as an emergency study for pandemic response. To select subjects, we utilized a multi-stage stratified random sampling method.

#### Sampling Method

**Figure 1:**
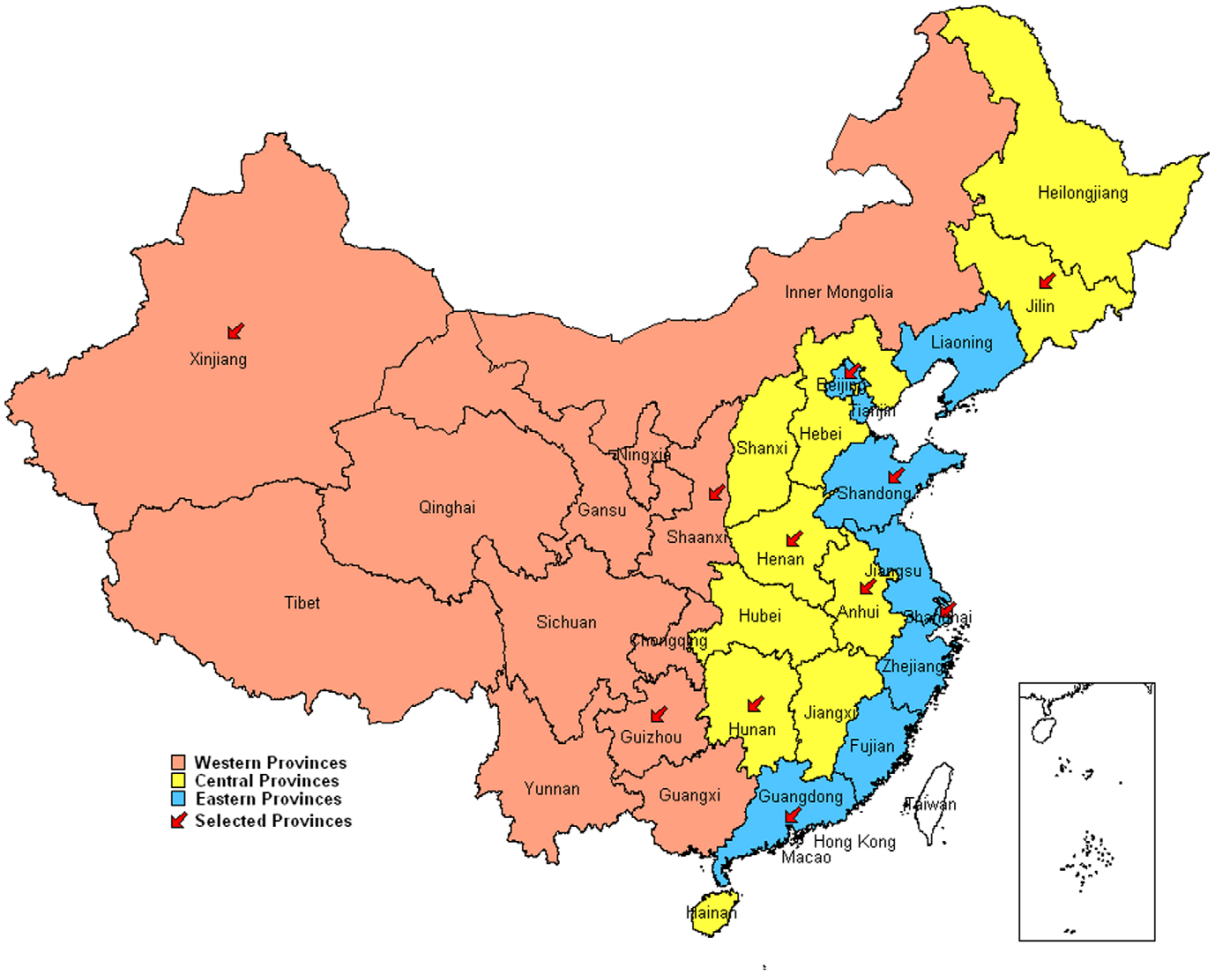
11 provinces selected randomly from eastern, central and western regions in the serological cross-sectional survey in January 2010. Western provinces include: Chongqing, Gansu, Guangxi, Guizhou, Inner Mongolia, Ningxia, Qinghai, Shaanxi, Sichuan, Tibet, Yunnan, and Xinjiang. Central provinces include: Anhui, Hainan, Hebei, Heilongjiang, Henan, Hubei, Hunan, Jiangxi, Jilin, and Shanxi. Eastern provinces include: Beijing, Fujian, Guangdong, Jiangsu, Liaoning, Shandong, Shanghai, Tianjin, and Zhejiang.

**Figure 2:**
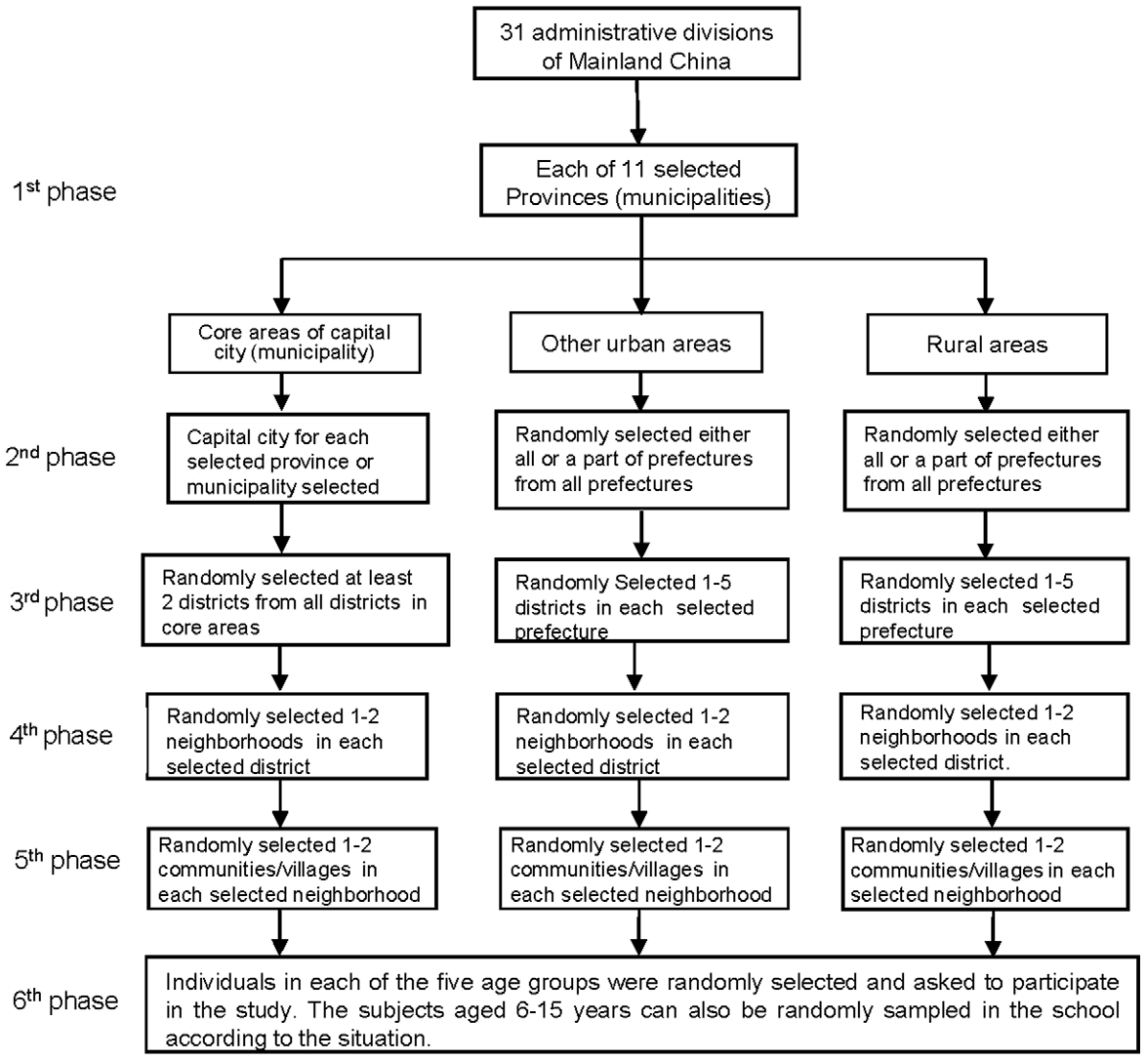
The sampling procedures in the serological cross-sectional survey in January 2010.

There are 31 administrative divisions of mainland China (22 provinces, 4 municipalities, 5 autonomous regions) that were divided into eastern, central and western regions by the National Bureau of Statistics of China. For this study, 12 provinces were randomly selected to participate. Four provinces (Beijing, Shandong, Shanghai and Guangdong) were randomly selected from eastern region, four provinces (Henan, Jilin, Anhui and Hunan) from central region, and four provinces (Shaanxi, Xinjiang, Guizhou and Tibet) from western region (Figure 1). Eleven of the twelve selected provinces agreed to conduct the study; Tibet declined to participate. The catchment population in the 11 provinces is 557.1 million, accounting for approximately 43% of the total population in mainland China. The remainder of the multi-stage random sampling method was carried out by each province. Each province was divided into three population strata, a) the core area of the capital city (municipality), b) prefectures of other urban areas and c) prefectures of rural areas. The provinces were then instructed to randomly select at least two districts in each of the three population strata, then 1–2 neighborhoods in each district and finally 1–2 communities/villages in each neighborhood (Figure 2). Once the communities were selected, sampling age groups for subjects 0–5 years, 6–15 years, 16–24 years, 25–59 years and ≥60 years were selected. Before the recruitment of participants, the team responsible for the site survey obtained a name list of all individuals (including age) residing in the communities/villages, and randomly selected individuals from each of age group. With the aid of community/village staff, the selected study subjects were approached and asked if they would like to participate in the study. Overall for each province, 300 persons from each age group in each of the three population strata was the target to enroll in the study. Selected subjects provided informed consent and could decline participation. If a selected individual declined to participate, the next individual on the list was contacted and asked to participate. If the informed consent was obtained from the study participant, the survey questionnaire was completed by a trained interviewer and blood samples were collected. For adults (≥18 years), the informed consent was provided by themselves. For adolescents (10–17 years), the assent was provided by themselves and the informed consent was provided by a parent or a legal guardian of the adolescent. For children (<10 years), the informed consent was provided by a parent or a legal guardian.

#### Sample Size

We expected the seroprevalence to be an estimated 25% and for a 95% confidence interval of +/− 10% (15–35%) we estimated the sample size of 300 from each age group in each of the three strata in each province for an expected sample size from each province of 4,500 and a total of 49,500 subjects.

### Investigation and specimen collection

From January 6–29, 2010, a standard questionnaire was administered by trained staff to subjects or guardians if the child was ≤15 years of age. Information collected on the survey included demographics (age, gender, occupation, etc.) and history of a cold, defined as any upper respiratory illness, since May 1, 2009, and pH1N1 vaccination history. The question describing occupation was asked of all individuals regardless of age and was classified as children in family care, children in kindergarten, student, teacher, doctor, and other. Children in family care are defined as the persons aged ≤15 years that do not fit into other categories such as children in kindergarten, students, or did not work in any other organizations or units described in the question. Blood samples were collected from each subject, 5 ml for subjects 6 years or older and 2–3 ml for children younger than 6 years. Serum samples were separated at prefectural Centers for Disease Control and Prevention (CDC) laboratories and then transported to the provincial CDC. After the conclusion of the study, the provincial CDC sent serum samples stored at -30°C and the survey database to the Chinese National Influenza Center (CNIC) of the National Institute for Viral Disease Control and Prevention (IVDC) at China CDC in Beijing for laboratory testing and data analysis.

### Laboratory testing

The haemagglutination-inhibition (HI) assay using 0.5% turkey red blood cells was used to test serum for antibody to pH1N1 according to standard protocols [27], [28]. The 2009 pH1N1 antigen used was the A/California/07/2009 virus (provided by U.S. CDC), which was propagated in specific pathogen-free (SPF) embryonated chicken eggs and inactivated with 1‰ paraformaldehyde. A positive serum control (SPF Chicken anti-serum against A/California/07/2009) and negative serum control (sera from health populations before the outbreak of pandemic H1N1) were included in each 96-well plate during the experiment. Prior to testing by the HI assay, serum samples were treated with a 1∶5 (vol/vol) of receptor destroying enzyme (RDE, prepared by CNIC) at 37°C for 18 hours followed by incubation at 56°C for 30 minutes. Serum samples were titrated in 2-fold dilutions in phosphate-buffered saline and tested at an initial dilution of 1∶10. Most individuals infected with influenza develop antibody titers ≥40 by viral HI assay after recovery [7] and was therefore used as marker for immunity against pH1N1 in this study.

### Laboratory Quality Control

National specialist groups were convened to guide statistical design, epidemiological investigation, laboratory testing, training and data analysis. Site supervisions by CDC at national or provincial level were conducted during the site investigation. All the villages were selected at the provincial level. Trained County CDC staffs were responsible for administering the questionnaire, collecting the blood specimens, and separating, storing and transporting the serum specimens. Bar code, material for sera collection and separation were provided by CNIC. Five-percent of serum samples were randomly selected from all samples were tested to assess the within-laboratory reproducibility. No more than 18% of replicate tests differed by more than 2-fold. The average reproducibility for positive and negative value is 92%.

### Statistical analysis

CNIC issued a standard database to all study sites, which was created in EPI Data software (version 3.02). The survey questionnaires were double inputted into the database and checked for consistency within the provinces. Data were analyzed at CNIC, with SAS 9.1 (SAS Institute, Cary, NC, U.S.) software.

### Applying weights to the data

Appropriate sampling weights were constructed for the national database and applied to seroprevalence data to account for the complex sampling design [29]–[31] and to adjust for age and community setting (capital, urban, rural) which were not representatively sampled. The weight components computed for these data were based on previously published weighting methodology which consisted of base weights and adjustment weights [29]–[31]. A base weight denotes the probability of selecting a participant from the total number of the sampling units of each sampling stage. Then adjustment weights were calculated to adjust seroprevalence of pH1N1 for differences between census characteristics of study sample and characteristics of the chinese population. The base weight of each sampling stage was calculated by dividing the total number of the sampling unit (e.g. province, capital city, district, neighborhood, village/community, and individual r) by the number of each sampling unit selected, described in Table S1. For example, the base weight for Guangdong Province on the first sampling stage was calculated by dividing the total number of eastern provinces (9) by the number of eastern provinces selected (4). The total base weight for a person (*i)* was then calculated by multiplying each of the base weights for each selected sampling unit including the person *i* on each sampling stage. Next the adjusted weights were calculated accounting for gender (male, female), age group (0–5 years, 6–15 years, 16–24 years, 25–59 years, and ≥60 years) and community type (capital city, urban area, or rural area). The adjusted weight of person *i* was calculated based on the combination of the three strata (age group, gender, and community type) that person *i* fit into by dividing the actual Chinese population for the combination by the sum of the base weights of all sampled individuals for the same combination of the strata that person *i* fell into, described in Table S2. The sampling weight of each selected individual was calculated by multiplying the total base weight by the adjustment weight.

To examine the association between risk factors and having a serological response to pH1N1 infection, we conducted multivariable logistic regression analyses. The dependent variable was presence of pH1N1 seropositivity vs. no seropositivity. Independent variables examined were gender, occupation, location of communities (capital city or rural areas vs. other urban areas), region (eastern or central vs. western). The final model examining risk factors for pH1N1 infection included gender, occupation, region, and location of communities (capital city or rural vs. other urban areas). Age group was excluded from the model because of the collinear relationship with occupation (p<0.0001). The surveyfreq procedure in the SAS software package was used to calculate the point estimates and 95% confidence intervals of weighted prevalence and the surveylogistic procedure was used for multivariable logistic regression to examine odds of infection for risk factors [32].

## Supporting Information

Figure S1 **Number of laboratory-confirmed pH1N1 cases and time when the serological cross-sectional survey conducted.**
(TIF)

Figure S2 **Geographical distribution of stored serum samples collected between 2006 and 2008.**
(TIF)

Table S1 **The calculation of base weights in each of 6 random sampling stage.** The base weight for person i can be expressed as follows: *W_basei = _ W_1_ ×W_2_×W_3_×W_4_×W_5_×W_6_*
(DOC)

Table S2
**The calculation of adjustment weights.** Adjustment weights (*Wadj*) were constructed based on post-stratification adjustments to account for the region, sex and age distribution of the entire Chinese population. If person *i* is located in the cell (row r, column c), his/her adjustment weight can be expressed as follows:
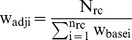
 N_rc_ refers to the actual size of the Chinese population in the cell (row *r, column c)*; n_rc_ refers to the sample size in the cell (row *r, column c)*; 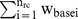 refers to the sum of base weights of all study individuals in the cell (row *r, column c).*
(DOC)
