# Supplementary figures and images for: The Seroprevalence of Pandemic Influenza H1N1 (2009) Virus in China
Source: PLoS One. 2011 Apr 21;6(4):e17919. doi: 10.1371/journal.pone.0017919 (PMC3080876; doi:10.1371/journal.pone.0017919)

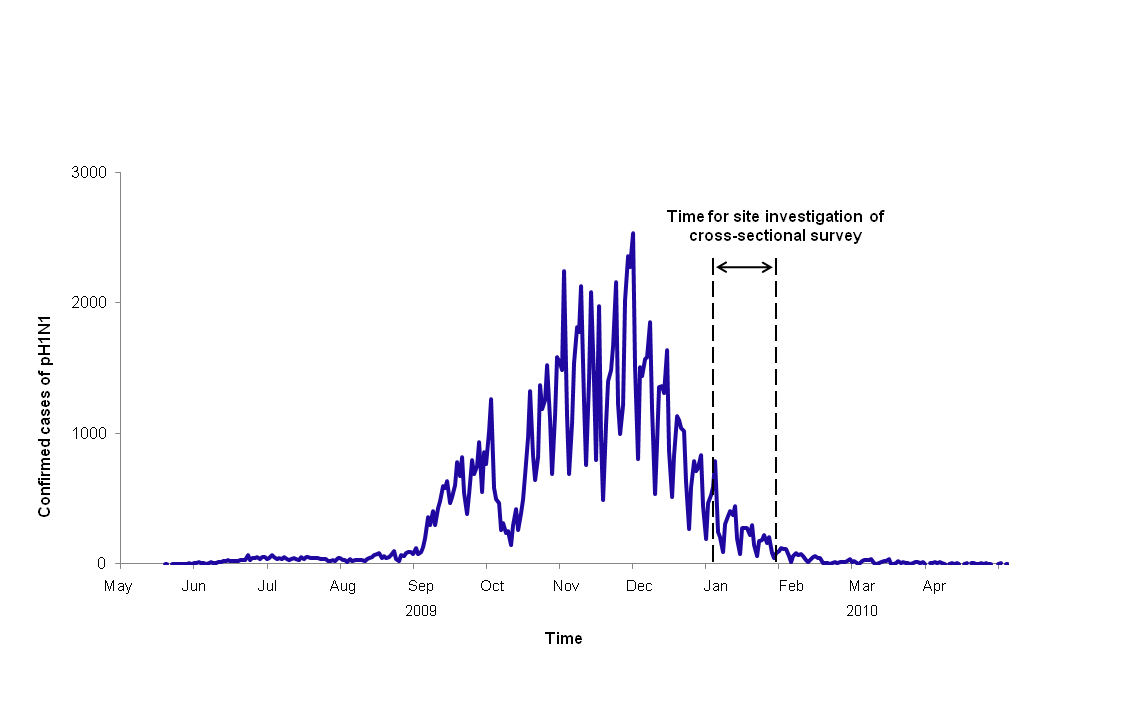

Supplement: Figure S1 — Number of laboratory-confirmed pH1N1 cases and time when the serological cross-sectional survey conducted. (TIF) [file pone.0017919.s001.tif]

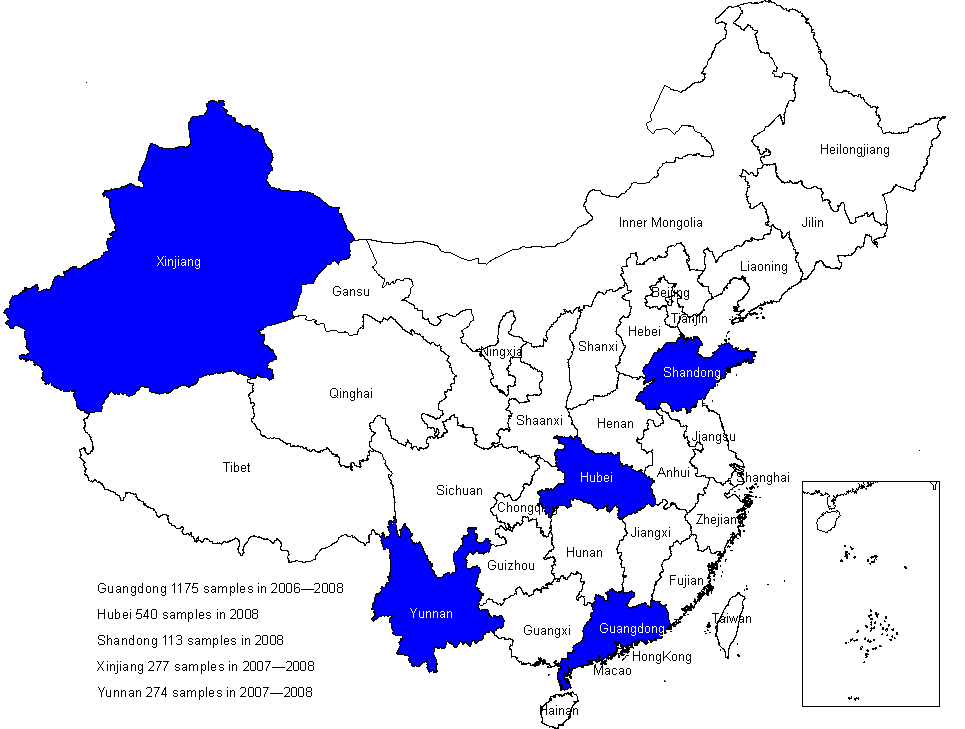

Supplement: Figure S2 — Geographical distribution of stored serum samples collected between 2006 and 2008. (TIF) [file pone.0017919.s002.tif]
