# Supplementary material for: The Seroprevalence of Pandemic Influenza H1N1 (2009) Virus in China
Source: PLoS One. 2011 Apr 21;6(4):e17919. doi: 10.1371/journal.pone.0017919 (PMC3080876; doi:10.1371/journal.pone.0017919)
Supplement: Table S1 — The calculation of base weights in each of 6 random sampling stage. The base weight for person i can be expressed as follows: Wbasei = W1 ×W2×W3×W4×W5×W6 (DOC) [file pone.0017919.s003.doc]

| Stage | Sampling unit | Base Weight (*W_base_*) |
| --- | --- | --- |
| 1 | Province | 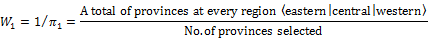 |
| 2 | Prefecture | 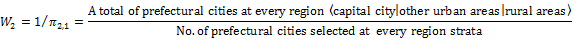 |
| 3 | District/County | 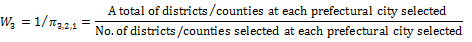 |
| 4 | neighborhood /Town | 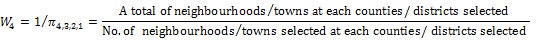 |
| 5 | community/ Village | 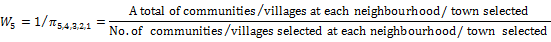 |
| 6 | Individual | 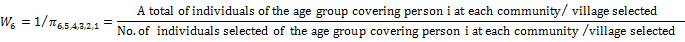 |
