# Supplementary material for: The Seroprevalence of Pandemic Influenza H1N1 (2009) Virus in China
Source: PLoS One. 2011 Apr 21;6(4):e17919. doi: 10.1371/journal.pone.0017919 (PMC3080876; doi:10.1371/journal.pone.0017919)
Supplement: Table S2 — The calculation of adjustment weights. Adjustment weights (Wadj) were constructed based on post-stratification adjustments to account for the region, sex and age distribution of the entire Chinese population. If person i is located in the cell (row r, column c), his/her adjustment weight can be expressed as follows: Nrc refers to the actual size of the Chinese population in the cell (row r, column c); nrc refers to the sample size in the cell (row r, column c); refers to the sum of base weights of all study individuals in the cell (row r, column c). (DOC) [file pone.0017919.s004.doc]

| Region | Male | | | | |  | Female | | | | |
| --- | --- | --- | --- | --- | --- | --- | --- | --- | --- | --- | --- |
|  | 0-5yrs | 6-15 yrs | 16-24 yrs | 25-59 yrs | ≥60 yrs |  | 0-5yrs | 6-15 yrs | 16-24yrs | 25-59yrs | ≥60 years |
| Capital city (Municipalities) | 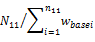 | 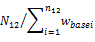 | … | … | 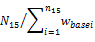 |  | 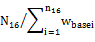 | 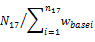 | … | … | 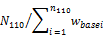 |
| Other urban areas | 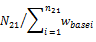 | 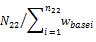 | … | … | 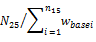 |  | 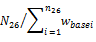 | 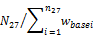 | … | … | 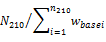 |
| Rural areas | 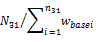 | 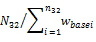 | … | … | 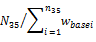 |  | 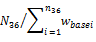 | 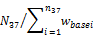 | … | … | 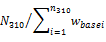 |
